# Intralesional Steroids for Alopecia Areata

**DOI:** 10.4103/0974-7753.66920

**Published:** 2010

**Authors:** M Kumaresan

**Affiliations:** Department of Dermatology, PSG Hospitals, Coimbatore, India

## INTRODUCTION

Alopecia areata (AA) is a type of autoimmune disease characterized by hair loss that has a variable presentation and course. Some patients exhibit spontaneous remission, while others progress to develop total loss of scalp and body hair. The current therapy for AA is not curative, but rather aimed at controlling or limiting the pathogenic process. Intralesional corticosteroids (ILCs) are used frequently in AA. Their use was first described in 1958, with the use of hydrocortisone.[1]

## MODE OF ACTION

Steroids with low solubility are preferred for their slow absorption from the injection site, promoting maximum local action with minimal systemic effect. Immunosuppression is the main mechanism of action.[2] Corticosteroids suppress the T-cell-mediated immune attack on the hair follicle. Preparations used include triamcinolone acetonide, triamcinolone hexacetonide, and hydrocortisone acetate. Triamcinolone acetonide is the preferred intralesional product because it is less atrophogenic than triamcinolone hexacetonide.[3,4]

## INDICATIONS

The efficacy of ILCs injection is variable depending on the patient population treated.[5] The efficacy appears to be greatest in certain groups of patients including those with less than 75% scalp hair loss, children, and those with a shorter duration of hair loss.[6] Patients with extensive AA, rapidly progressive disease, and greater than two years’ duration of the current episode, respond poorly to ILCs.[7] Patients with exclamation point hairs and a positive hair pull test respond better to ILCs, as these reflect the active inflammation of the follicles.[5] It is difficult to evaluate the efficacy of ILCs in AA, as it is known to show spontaneous remission.[5]

## EVIDENCE FROM LITERATURE

Although ILCs have been used in the treatment of AA for about 50 years, there are no published randomized controlled trials.[8,9] Porter and Burton[10] showed that hair regrowth was possible in 64 and 97% of AA sites treated by intralesional injections of triamcinolone acetinoide and its less-soluble derivative, triamicinolone hexacetonide, respectively. Abell and Munro reported that 52 of 84 patients (62%) showed regrowth of hair at 12 weeks after three injections of triamcinolone acetonide, using the Porto Jet needleless device, compared to one of 15 (7%) control subjects injected with isotonic saline.[7] Chang *et al*,[5] had reported that six out of 10 patients, with AA involving more than 50% of the scalp, responded favorably to ILCs. An uncontrolled study from Saudi Arabia found 63% of the patients receiving monthly triamcinolone injections showed complete regrowth.[11]

## METHOD OF ADMINISTRATION

ILCs preferably triamcinolone acetonide is the first-line therapy for adult patients with less than 50% of scalp involvement.[2,4,12] Concentrations of 2.5 to 10 mg/mL may be used, but 5 mg/mL (maximum volume of 3 mL per session) is the preferred concentration for scalp.[2–4,12] For the eyebrows and face, 2.5 mg/mL can be used (0.5 mL to each eyebrow).[12] A concentration of 10 mg/mL with a maximum total of 2 mL, or 5 mg/mL for a maximum total of 4 mL, has also been reported for use on the scalp, at one visit.[3] Triamicinolone acetinoide is injected intradermally with a 0.5-inch long, 30-gauge needle, as multiple 0.1-mL injections at 1-cm intervals.[12] BD insulin (1 cc) syringes are a good choice, due to lack of leak between syringe and needle. Sterile saline is preferred over Xylocaine as a dilutent, because the latter stings more.[3] Optional topical anesthetic can be applied 30 to 60 minutes before treatment to minimize pain from the injections, this will be useful when treating eyebrows.[3,5,12] ILCs may also be administered by a needleless device (e.g. Dermajet™). The device should be sterilized between patients.

Treatments are repeated every four to six weeks.[3,4,9,12] Initial regrowth is often seen in four to eight weeks.[2,3,12] If there is no improvement after six months of treatment, the ILCs should be stopped. The decreased expression of thioredoxin reductase 1 in the outer root sheath may be the cause for glucocorticoid resistance in some AA patients.[13,14] Children younger than 10 years are not usually treated with ILCs because of pain localized at the injection sites.[2,3,12]

## ADVERSE EFFECTS

**Figure 1:**
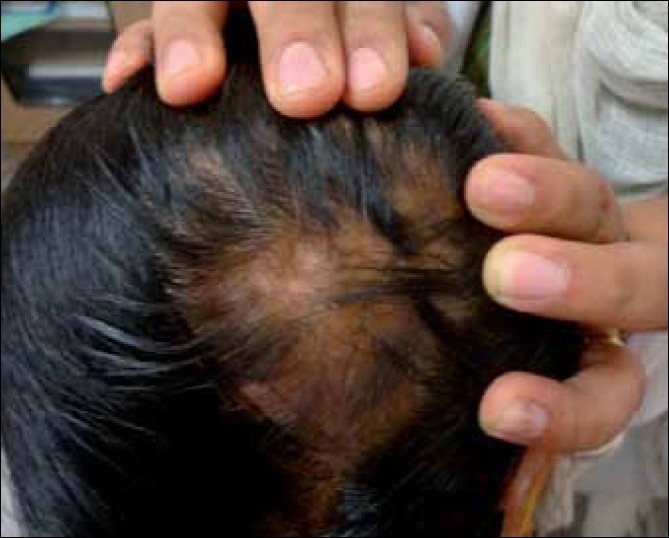
ILCs-induced atrophy on scalp

**Figure 2:**
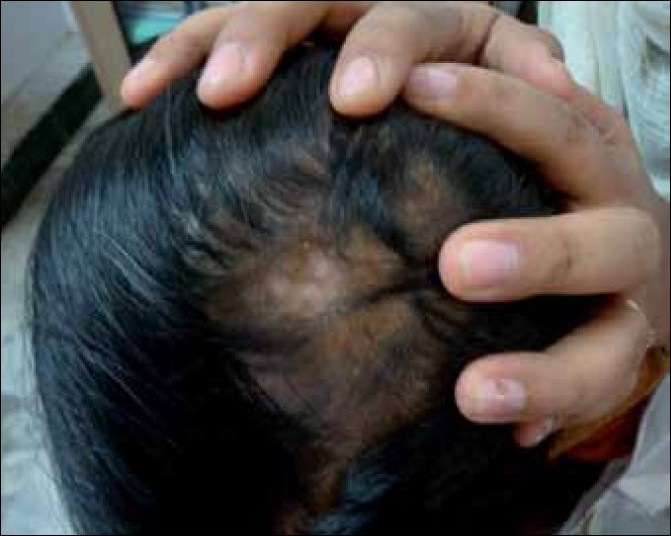
ILCs-induced depigmentation on scalp

The common adverse effects noted during ILCs therapy are, pain, atrophy of skin and hair follicles [Figure 1], telangiectasia, hypo / depigmentation [Figure 2] and cushingoid features, due to systemic absorbtion.[2–5,12] The main side effect is minimal transient atrophy.[2] This can be prevented by avoiding injections that are too great in volume per injected site, too frequent or too superficial (intra-epidermal).[2] The presence of atrophy should not lead to discontinuation of the treatment. Re-injecting areas of denting, if avoided, is sufficient to allow the atrophy to revert. The ultrasonic assessment of cutaneous atrophy by ILCs has revealed that the atrophy is transient and eventually normal thickness of the skin is regained.[15] Transient follicular atrophy has been reported with higher concentrations of ILCs.[5] There is a risk of cataract and raised intraocular pressure if ILCs are used close to the eyes, for example, when treating eyebrows.[16] There is a single case report of anaphylaxis in a patient receiving intralesional triamcinolone acetonide for treatment of Alopecia Areata.[17]

## CONCLUSION

ILCs are most suitable for patchy, relatively stable hair loss, of limited extent. This modality is not appropriate in rapidly progressive AA or in alopecia totalis / universalis. ILCs are still the preferred method of treatment for most patients.

Key points

1. ILCs preferably triamcinolone acetonide is the first-line therapy for adult patients with less than 50% scalp involvement.
2. The preferred concentration for the scalp is 5 mg/mL and for the face and eyebrows it is 2.5 mg/mL.
3. BD insulin (1 cc) syringes are a good choice, due to lack of leak between syringe and needle.
4. If there is no improvement after six months of treatment, ILCs should be stopped.
5. Atrophy can be prevented by avoiding injections that are too great in volume per injected site, too frequent or too superficial.
